# The Effects of Exercise on Aging-Induced Exaggerated Cytokine Responses: An Interdisciplinary Discussion

**DOI:** 10.1155/2022/3619362

**Published:** 2022-01-23

**Authors:** Soontaraporn Huntula, Laddawan Lalert, Chuchard Punsawad

**Affiliations:** ^1^Department of Sport and Exercise Science, School of Medicine, Walailak University, Nakhon Si Thammarat 80160, Thailand; ^2^Department of Medical Science, School of Medicine, Walailak University, Nakhon Si Thammarat 80160, Thailand; ^3^Research Center in Tropical Pathobiology, Walailak University, Nakhon Si Thammarat 80160, Thailand

## Abstract

Aging is generally known to be associated with dynamic biological changes, physiological dysfunction, and environmental and psychological decline. Several studies have suggested that aging is associated with increased inflammatory cytokines, causing several diseases. However, the effect of exercise on aging has been less delineated, and the relationships between cytokine activation, aging, and exercise also need further study. Here, we discuss some ideas about the effect of exercise on aging-induced exaggerated cytokine responses and discuss the possible roles of the aging-induced exaggerated cytokine response following exercise. Evidence from these findings suggests that exercise is a beneficially applicable model to use in studies on the mechanisms underlying the age-associated gradated cytokine response, and these results may provide guidelines for health professionals with diverse backgrounds.

## 1. Introduction

Cytokines are a group of secreted proteins induced by specific immune molecular messengers that can regulate and mediate more than one pathway; many act in feedback loops to control their own production paradoxically at different times [1]. However, it seems possible that compared with other body systems, homeostatic regulation and modulation of the immune response become more fragile and less tightly focused during increasing age [2]. The imbalance between proinflammatory and anti-inflammatory cytokine levels is a common characteristic of both aging and aging-related diseases [3]. However, a lack of exercise is one of the reasons associated with deconditioning, fatigue, onset of disease, and an increase in inflammatory cytokines [4]. Furthermore, cytokine and inflammatory activity are increased by aging [5], whereas exercise can act against all these situations, although the relationship of these variables is extremely complex and dependent on several factors [6]. Therefore, this review aims to examine the relationship of exercise with cytokine changes in aging. This review provides guidelines for health fitness in working with an aging population.

## 2. Exercise Formulation

Exercise is a physical activity that enhances and maintains physical fitness. Exercise has both beneficial and harmful effects on performance cognition, and there seems to be improvement by engaging in acute exercise at a moderate intensity. Several studies have suggested that exercise plays a role in the prevention of some cancers [7] and reduces the risk of heart disease, hypertension, osteoporosis, obesity, type II diabetes, osteoarthritis, and abnormal cholesterol [8, 9]. Moreover, exercise improves overall immune function, which is important for seniors, as their immune systems are often compromised [10, 11]. However, to benefit from exercise, the extent or intensity of exercise for various life stages is still uncertain.

For the aging population, the risk of exercise needs to be considered for individual persons with limited factors. Generally, both warm up and cool down are important phases to simultaneously increase muscle and functional range of motion before and after exercise, respectively. With an aging population that is sedentary, a progressive low-intensity aerobic exercise program is recommended [12]. Gradual increases in duration and intensity are encouraged. In movement activities such as group-led aerobics, there should be concern regarding sudden movement changes and elaboration on choreography that may lead to falls. Some activities such as running and jogging may unnecessarily cause stress to the knees and hips; thus, these activities are not universally recommended for the aging population [13]. Additionally, resistance training programs have demonstrated some clear and consistent results, showing that elderly individuals 67 to 91 years of age can significantly improve muscular strength, functional mobility, and balance [14, 15]. Additionally, regarding the cardiovascular and musculoskeletal systems of the aging population, there appear to be relatively few contraindications to strength training [16]. Resistance training may not be encouraged for some hypertensive individuals due to elevated blood pressure [17]. From the results of the research cited, all types of exercise are strongly recommended for aging persons, with consideration of individual factors and limitations.

## 3. Inflammatory Cytokine Production

Cytokines are a variety of hormone-like polypeptide mediators that regulate inflammation and immune responses in intercellular signaling systems [18]. These signaling molecules usually act in an autocrine or paracrine manner to induce a physiological protective response to initial tissue injury [19].

Tumor necrosis factor alpha (TNF-*α*), interleukin 1 beta (IL-1*β*), and interleukin 6 (IL-6) are important proinflammatory cytokines that are considered the main cytokines in the pathogenesis of sepsis [6, 20]. TNF-*α* seems to be the first cytokine released when systemic inflammation is observed and shows high levels within several hours after the onset of sepsis, followed by IL-1 and then IL-6 [21]. These proinflammatory cytokines are promoted after acute inflammatory responses, such as leukocytosis (neutrophilia), by inducing chemokines such as IL-8 and monocyte chemotactic protein- (MCP-) 1 [22]. It is considered a “master regulator” of the innate immune response based on studies demonstrating that TNF‐*α* blockade directly influences the production of a variety of other pro- and anti-inflammatory factors, such as IL-1, IL-6, IL-8, GM-CSF, and adiponectin [23]. Furthermore, TNF-*α* is an inflammatory cytokine that works together with IL-1*β* to produce anorexia, induce lipolysis, and then lead to apoptotic cell death [24]. Moreover, TNF-*α* can stimulate IL-6 release, which upregulates protein degradation. Previous studies have suggested that TNF‐*α* is involved in age-related neurodegenerative diseases, such as Alzheimer's disease (AD). For example, the level of TNF‐*α* in cerebrospinal fluid is increased 25-fold in AD patients relative to healthy controls and correlates with clinical decline [25]. In addition, the expression of single-nucleotide polymorphisms within the promoter region of the TNF-*α* gene increased the risk for AD [26].

IL-6 is an important cytokine that is correlated with the aging process, which leads to muscle decrease and loss of bone mass [27]. IL-6 is released from macrophages and T lymphocytes. Both TNF-*α* and interleukin 1 beta (IL-1*β*) promote the vigorous release of IL-6, whereas IL-6 can be released in a feedback mechanism to degrade the receptors for and the release of TNF-*α* and IL-1*β*, yielding an anti-inflammatory effect [28]. In the aging population, progressive IL-6 release has been shown to have cross-effects associated with muscle, strength, performance, and balance [29]. In addition, most studies have focused on IL-6, which plays an important role in a wide range of immunological, inflammatory, and metabolic functions [30, 31]. However, the concentration of IL-6 seems to be increased by the incidence of age-related diseases [32]. Herein, the exacerbated production of IL-6 is associated with age-related diseases, suggesting that IL-6 plays a pathogenetic role in age-related diseases such as osteoporosis, AD, multiple myeloma, or atherosclerosis [30].

IL-1*β* is generally known as a proinflammatory cytokine. The action of this cytokine not only activates its own release but can also induce TNF-*α*. IL-2 activation by stimulating T helper cells promotes tissue inflammatory activity [6]. Moreover, IL-1*β* can be argued to both stimulate the cascade of inflammatory functions and trigger increased amyloid protein in the development of AD [33].

In summary, inflammatory cytokines are activated and enhanced due to aging-related conditions associated with several diseases in the aging population, such as cognitive decline, loss of muscle, osteoporosis, and immune dysfunction. The roles of anti-inflammatory cytokines are very interesting issues to be addressed in this regard.

## 4. The Roles of Cytokines in Aging Diseases

Cytokine dysregulation is believed to play a key role in the remodeling of the immune system at older age, which seems to be a marker of unsuccessful aging. Neuroinflammation is increasingly implicated in age-related neurodegenerative diseases, with a progressive tendency toward a proinflammatory phenotype called “inflammaging,” such as AD and Parkinson's disease (PD). Here, we discuss the major age-related diseases that appear to contribute to immune imbalance and cytokine dysregulation, which is associated with “inflammaging” or para-inflammation.

### 4.1. Alzheimer's Diseases

Alzheimer's disease (AD) is the most common neurodegenerative disorder, and its neuropathological hallmarks are *β*-amyloid (A*β*) plaques and neurofibrillary tangles [34]. The inflammatory process has a fundamental role in the pathogenesis of AD and is related to AD neuroinflammation, including brain cells, such as microglia and astrocytes, the complement system, cytokines, and chemokines. An important factor in the onset of the inflammatory process is the overexpression of IL-1, which produces many reactions in a vicious cycle, leading to dysfunction and neuronal death [35]. Other important cytokines in neuroinflammation are IL-6 and TNF-*α* influencing the surrounding brain tissue by activated microglia, which may play a potentially detrimental role by eliciting the expression of proinflammatory cytokines. Microglia activated by lipopolysaccharide (LPS), IFN-*α,* or TNF-*α* are considered to be the “M1” (“classically activated”) form of microglial cells. M1-skewed microglial activation plays a vital role in the defense against pathogens and tumor cells by producing proinflammatory cytokines, such as IL-1*β*, TNF-*α*, STAT3, IL-6, IL-12, and IL-23, and free radicals, such as reactive oxygen species (ROS) [36]. Conversely, the alternative M2 anti-inflammatory phenotype promotes tissue repair and angiogenesis by releasing high levels of anti-inflammatory cytokines such as IL-10, IL-4, IL-13, and TGF-*β* and low levels of proinflammatory cytokines [37].

### 4.2. Parkinson's Diseases

Parkinson's disease (PD) is one of the most common neurodegenerative diseases of aging in the world. PD is the second most common neurodegenerative syndrome after AD, affecting 1-2% of the population aged 60 years [38]. Clinical research has revealed that neuroinflammation may play an important role in the death of dopaminergic neurons in the substantia nigra, which is the pathological hallmark of PD [39, 40]. Cytokines, including IL‐1, IL‐6, IL‐10, IFN‐*γ*, transforming growth factor- (TGF-) *β,* and TNF‐*α*, are the most investigated cytokines for PD. Previous studies found that the expression of TNF‐*α* was higher in the striatum and substantia nigra of 6-OHDA-induced PD model rats than in control rats, which links the substantia nigra and the striatum. TNF‐*α* expression was upregulated, which was accompanied by microglial activation [41]. Furthermore, IL‐1 is a cytokine that is described as an inflammatory factor and neurotoxic mediator. The secretion of IL‐1*β* has a similar proinflammatory effect, which can be reversed by specific IL-1 inhibition. An IL-1 receptor antagonist significantly attenuates the augmented loss of DA neurons caused by lipopolysaccharide (LPS) or 6-OHDA-induced sensitization to DA degeneration [42]. In addition, IL-6 is a common proinflammatory cytokine; however, it is not clear whether IL‐6 also has a significant role in PD. Scalzo et al. found that serum levels of IL-6 were markedly increased in patients with PD and that PD patients who exhibited higher serum levels of IL-6 demonstrated a reduced gait speed, more problems in performing tasks that required postural changes and reduced base support, indicating that IL-6 may be associated with motion function in patients with PD [43].

### 4.3. Multiple Sclerosis

Multiple sclerosis (MS) is an autoimmune-associated human central nervous system (CNS) disorder. The pathogenesis of the human demyelinating disorder MS is a key facet that predominates despite conjecture over the precise involvement of the disease [44], which involves the loss of immune tolerance to self-neuroantigens. Moreover, evidence verified a prematurely aged immune system that may change the frequency and profile of MS through an altered decline in immune tolerance. Immune aging is closely linked to chronic systemic suboptimal inflammation, termed inflammaging (IFA), which disrupts the efficiency of immune tolerance by varying the dynamics of immunosenescence (ISC), which includes accelerated changes to the immune system over time [45]. In particular and fundamentally, there is a primary breakdown of peripheral T cell tolerance to myelin-associated antigens preceding an enduring, immune cell-driven inflammatory pathology that is responsible for the characteristic demyelination, and neurodegeneration that causes gradual and varying disability [46–48]. However, a decline in immune tolerance coupled with the consequences of an aging immune system will have a cumulative and enduring effect on the development and progression of diseases with an autoimmune etiology. In addition, MS has an emerging, progressive, and distinctive pathophysiology with typical clinical profiles that allows ultimate diagnosis of a neurodegenerative CNS disease [49].

## 5. Cytokine Changes That Occur with Aging

It is now established that cytokines are an essential part of pathophysiology. It is well known that the symptoms and recovery from illness caused by brain damage and infections become more severe [19]. Several cytokine categories have been discovered, including chemokines, lymphokines, interleukins (ILs), interferons (IFNs), transforming growth factor (TGF), and tumor necrosis factor (TNF). Proinflammatory cytokine production is an initial active process that is followed by anti-inflammatory cytokine activation [50]. Furthermore, cytokine dysfunction is considered to play a vital role in the immune system during the aging period, with evidence suggesting an inability to control the inflammatory system, which seems to be a key point of unsuccessful aging [3]. Both age-related and low-grade inflammation have been associated with the aging process by exaggerating or enhancing proinflammatory cytokines [51]. In aging, the release of proinflammatory cytokines, such as IL1, IL6, and TNF-*α,* activates peripheral blood mononuclear cells (compared with those in young individuals) by upregulating the proinflammatory cytokine response.

These age-associated changes are speculated to be related to the exacerbated cytokine response [52]. In biological systems, aging has numerous impacts on molecular and cellular damage over time. These impacts lead to a decrease in physical activity and mental health, an increased risk of several diseases, and death. Aging can be defined as a general loss of physical activity that involves the interaction of both physiological and behavioral factors. It is widely acknowledged that aging is associated with body function and activity losses rather than gains [53]. Generally, when people grow older, many chronic and systemic diseases, such as cardiovascular diseases, diabetes type 2, cancerous tumors, pulmonary disease, musculoskeletal diseases, falls, and neurological and cognitive dysfunction, could plausibly develop [54]. As a result, aging is associated with an increase in inflammatory activity, whereas the plasma concentrations of proinflammatory cytokines are very low in healthy individuals, corresponding to the general concept that aging induces enhanced neuroinflammation [5]. In a previous study, we also found that in primary culture, aging microglial M1 transition (*neuroinflammatory M1 microglia*) was increased, but the M2 transition (*neuroprotective M2 microglia*) was diminished by aging, confirming the general sense that aging induces neuroinflammation. Primary cultures of microglial cells from aged mice tend to adopt an M1 activation phenotype, whereas IL-4-induced Arg1 expression is attenuated in the microglia of aged mice [55]. Thus, aging is associated with prolonged inflammatory phenomena and has also been an important factor in the pathogenesis of age-related diseases. Accordingly, further studies of aging microglia are important for a better understanding of the roles, phenotype, and gene expression in both aging and neurodegenerative diseases.

## 6. Benefits of Exercise for Aging

As mentioned above, aging has been found to be correlated with a prolonged inflammatory response and is a key factor in the pathogenesis of age-related diseases. Aging is associated with various negative outcomes, including cognition, falls, fear of falling, hospitalization, increased healthcare utilization, polypharmacy, and mortality [56]. There are clearly many benefits that could be gained from exercise. A previous study suggested that exercise is generally believed to enhance and maintain physical fitness and overall health and wellness (including cardiovascular disease, neuropsychological function, osteoporosis, glucose metabolism, diabetes, and functional ability) and reduce the risk of heart disease and osteoarthritis [57–59]. Additionally, exercise has underlying effects on cognition and behavior involving neuronal structure and responsiveness in juvenile and adult animals. For example, enhanced long-term potentiation in exercising adult Sprague–Dawley rats has been correlated with increased mRNA expression of the NR2B subunit of the *N*-methyl-D-aspartate receptor in the dentate gyrus, which may cause enhanced learning ability due to exercise [60]. Moreover, the resistance training program applied to an elderly population for six months showed a tendency to reduce the frequency of cells with micronuclei (∼15%) and the total number of micronuclei (∼20%), leading to improved resistance against genomic instability [61]. These findings suggested that exercise decelerates the aging process in individuals, which decreases the risk of moderate or severe functional limitations in aging societies.

Moreover, IL-6 is produced in larger amounts than any other cytokine in relation to exercise. Kohut and colleagues suggested that IL-6 increases in the generation of the acute phase response after exercise for 45 min. Increased levels of IL-6 and IL-18 were found immediately after an aerobic exercise class at up to 65–80% VO_2_ max for 3 days per week but not after flexibility/resistance exercise [62]. On the walking at the rate of perceived exertion (RPE) scale, the IL-6 level in blood has been shown to be significantly decreased for 150 min per week after walking exercise at RPE 12-13 and resistance training at RPE 15-16 [63].

### 6.1. Benefits of Exercise for Cardiovascular Disease and Hypertension on Aging

Previous research has provided evidence of the development of the major risk factors for age-related diseases, including cardiovascular disease, obesity, diabetes, and hypertension [64]. The high prevalence of both cardiovascular disease and hypertension in modern industrialized societies imposes a considerable public health problem [65]. However, hypertension is a major and serious health problem that increases the risk of coronary heart disease [66].

Cardiovascular disease is the leading cause of death among elderly individuals. Aging is an inevitable part of life and the largest risk factor for cardiovascular disease and is associated with a steady decline in physiological processes. This leads to an increased risk of health complications and disease by delivering oxygenated blood to all tissues in the body. Aging has a profound effect on the heart and blood vessels, which leads to an increase in cardiovascular disease [67]. However, regular exercise can improve cardiovascular health by affecting the coagulation of blood in the blood vessels. It can reduce cardiovascular disease risk factors by stimulating the environment by being anti-inflammatory (through the release of myokines coming from the muscle), promoting the regeneration of the heart muscle, and alleviating age-related loss of muscle mass and strength [68]. Regular exercise resulted in a slower decrease in peak oxygen uptake, better left ventricular and diastolic function, and decreased arterial stiffness. The ability to predict cardiovascular risk factors in young and middle-aged adults declines with advancing age. Factors in the elderly remained helpful. Exercise training in both the normal and hypertensive groups between the ages of 60 and 79 was shown to decrease systolic and diastolic blood pressure down from 6 to 9 mm Hg. A small number of intervention studies suggest that exercise results in a slight increase in high-density lipoprotein (HDL) cholesterol (4% to 9%) without a significant change in HDL cholesterol. [69].

Hypertension is a chronic inflammatory state and a major risk factor for cardiovascular diseases. Previous studies suggested that proinflammatory cytokines such as TNF-*α* and IL-1*β* were increased within the hypothalamic paraventricular nucleus of spontaneously hypertensive rats [70], leading to the development of hypertension symptoms [71]. Physical inactivity has been shown to be associated with hypertension in epidemiologic studies [72]; hence, physical activity has been recommended in the prevention and treatment of hypertension [73]. Recently, exercise and physical activities have been reported to be beneficial for patients suffering from hypertension [74]. *In vivo* studies, the effect of exercise training on the production of proinflammatory cytokines and the anti-inflammatory cytokines TNF-*α*, IL-1*β*, IL-6, IL-10, and monocyte chemokine protein-1 (MCP-1) was measured in the hypothalamic paraventricular nucleus. The hypothalamic paraventricular nucleus levels of TNF-*α*, IL-1*β*, IL-6, and MCP-1 were higher than those in Wistar-Kyoto rats. Hence, exercise training reduced the hypothalamic paraventricular nucleus levels of TNF-*α*, IL-1*β*, IL-6, and MCP-1 in spontaneously hypertensive rats. Exercise training restored the balance between pro- and anti-inflammatory cytokines in the hypothalamic paraventricular nucleus of spontaneously hypertensive rats [75]. Moreover, many studies have provided evidence that resting systolic and diastolic blood pressures can be reduced from 10.8 to 8.2 mmHg, respectively, in mild hypertension patients when they perform physical activity [76]. Hence, high levels of physical fitness can reduce blood pressure among men and women [77, 78]. Brandon and colleagues reported the effects of a 4-month strength training program on strength and blood pressure in aging. The results revealed that systolic blood pressure remained unchanged, whereas mean arterial blood pressure and diastolic blood pressure decreased by 2.4 and 3 mmHg, respectively. Thus, moderate intensity strength training greatly improved strength in older adults and had no adverse effect on blood pressure responses [79]. Thus, exercise is strongly recommended for aging for several reasons, such as increasing muscle mass and reducing the risk of diseases. Importantly, a decrease in inflammatory cytokines exerts an anti-inflammatory effect and the beneficial effects of exercise on cardiovascular disease and hypertension in different age populations, as mentioned in Tables 1 and 2. However, the effects of exercise vary depending on the intensity, frequency, and duration of exercise or physical activity. The individual's characteristics need to be of concern, especially aging population. Additionally, we have provided the deleterious of exercise or physical activity on cytokine in different age populations in Table 3. This information may provide guidelines for exercise or physical activity with diverse backgrounds.

## 7. Conclusion

Exercise can moderate cytokine production in aging. In the aging population, exercise has been demonstrated to prevent disease, lower the risk of falls, improve mental health and well-being, strengthen social ties, and improve cognitive function. Many studies have reported lower levels of inflammatory cytokines in the active aging population than in the inactive aging population. However, insight into both what does or does not work regarding exercise on aging-induced exaggerated cytokine responses is not sufficient at present, and more research is necessary regardless of positive or negative results. These findings may not only help to reduce illness and promote recovery but also lead to new studies elucidating the defenses against stress and infection in the aging population.

## Figures and Tables

**Table 1:** Observational studies of the relationship between exercise and inflammatory cytokines in aging.

Age (years)	Exercise/physical activity	Inflammatory markers	References
74.3 ± 2.7	Moderate or strenuous activity (hand-grip strength, chair stands, and gait speed)	↓ CRP, ↓ IL-6	D.R. Taaffe et al. 2000 [80]
70–79 years old	Recreational activity, house/yard work activity, work activity, and total physical activity	↓ CRP, ↓ IL-6	D. B. Reuben et al. 2003 [81]
40–75 years old	Metabolic equivalent-hours (MET-hours) method	↓ sTNF-R1, ↓ sTNF-R2, ↓ IL-6, ↓ CRP	T. Pischon et al. 2003 [82]
70–79 years old	Physical activity questionnaire (MET-hours/weeks) physical activity from the following categories (housework, stair climbing, walking for exercise, other types of walking, aerobics/calisthenics, weight training, high-intensity exercises, moderate-intensity exercises, or work/volunteer/caregiving activities)	↓ CRP, ↓ IL-6, ↓ TNF-*α*	L. H. Colbert et al. 2004 [83]
48 ± 12	Physical activity (MET-hour/weeks)	↓ CRP, ↓ WBC count, ↓ TNF-*α*, ↓ IL-6	C. Pitsavos et al. 2004 [84]
63 ± 10	Maximal exercise testing on a motor-driven treadmill or an electrically braked supine bicycle	↓ CRP	K. Rahimi et al. 2005 [85]
70.3 ± 4.1 69.8 ± 5.5	Aerobic exercise treatment (cardio) or a flexibility/strength exercise treatment (flex) 3 days/week, 45 min/day for 10 months	↓ IL-18, ↓ IL-6, ↓ CRP	M. L. Kohut et al. 2006 [62]
18–65 years old	Physical activity test performed on a modified Monark cycle ergometer with 6 min workloads, each separated by a 1 min rest	↓ CRP, ↓ TNF-*α*, ↔ IL-6, ↔ adiponectin	B. J. Arsenault et al. 2009 [86]
76.4 ± 4.1 77.0 ± 4.4	Walking for 150 min per week at RPE 12–13 and resistance training at RPE 15-16	↓ IL-8, ↔ TNF-*α*, ↔ sTNFr I, ↔ sTNFr II, ↔ IL-6sR, ↔ IL-1sRII, ↔ IL-1ra	K. M. Beavers et al. 2010 [87]
>50 years old	70% of one maximum repetition (1 RM) or less was classified as moderate intensity, while activities requiring over 70% of 1 RM were classified as vigorous intensity	↓ CRP, ↓ IL-6, ↔ TNF-*α*	A. V. Sardeli et al. 2018 [88]

Notes: ↓ refers to significant decreased; ↔ refers to no change. CRP, C-reactive protein (CRP); IL-6, interleukin 6; sTNF-R1, soluble tumor necrosis factor receptor 1; sTNF-R2, soluble tumor necrosis factor receptor 2; TNF*α*, tumor necrosis factor alpha; WBC count, white blood cell count; IL-18, interleukin 18; sTNFr I, soluble tumor necrosis factor receptor 1; sTNFr II, soluble tumor necrosis factor receptor 2; IL-6sR, soluble interleukin 6 receptor; IL-1sRII, soluble interleukin 1 receptor 2; IL-1ra, interleukin 1 receptor antagonist.

**Table 2:** Observational studies of the beneficial effects of exercise on cardiovascular disease and hypertension in different age populations.

Age (years)	Aging effects	Exercise/physical activity	Beneficial of exercise/Physical activity	References
Cardiovascular disease				
64 ± 7.1 (coronary heart disease patient)	↑ CRP ↑HDL cholesterol ↑ LDL cholesterol ↑ Triglycerides	12-week aerobic exercise training program at 70–80% of individual maximal heart rate (continuous exercise on treadmill, stationary bicycle, arm bicycle, rowing machine, or a combination of these activities for 40 min/time)	↓ CRP ↓ HDL cholesterol ↓ LDL cholesterol ↓ Triglycerides	E. Goldhammer et al. 2005 [89]
45.44 ± 11.26 (heart failure patient)	↑ IL-6 ↑ sTNFR1	3–7-day interval with (1) moderate-intensity walk, with 60% of the peak heart rate achieved at the CPET in 30 min (M30) (2) low-intensity walk, with 40% of the peak heart rate achieved at the CPET heart rate corresponding to 40% of VO_2_ peak in 30 min (L30)	↓ IL-6 (1 h after exercise for L30) ↓ sTNFR1 (1 h after exercise for M30)	G. A. Ribeiro-Samona et al. 2017 [90]
>18 years old (cardiovascular disease)	↔ IL-1*β* ↑ IL-6 ↑ TNF-*α* ↔ IL-8 ↑ IL-25 ↑ Adam17	Five daily sessions of cycling for 3 weeks Each session was performed at 70% HR_max_ for at least 30 minutes (the sessions duration increased by ten minutes every three days, up to 50 minutes twice a day)	↔ IL-1*β* ↓ IL-6 ↓ TNF-*α* ↔ IL-8 ↓ IL-25 ↓ ADAM17	V. Racca et al. 2020 [91]

Hypertension				
67.8 ± 4.3 (aerobic group, AG) 67.8 ± 5.2 (resistance and aerobic group, RAG) 69.9 ± 5.5 (control group, CG) (hypertension)	↑ IL-6 ↑ TNF-*α*	Training lasted 10 weeks, with sessions held three times a week (AG: treadmill test with 50–70% VO_2max_) (RAG: trained with intensities of 50–60% 1RM; (1) leg press 45°, (2) chest press, (3) leg extension, (4) lat pull down, (5) leg curl, (6) pulley upright row, (7) seated calf raise, (8) machine seated row, (9) abdominal crunches)	↓ IL-6 (AG, RAG) ↓ TNF-*α* (RAG)	L. G. Lima et al. 2015 [92]
76.8 ± 4.7	↑ GDF-15	6-minute walk distance over 12 months	↓ GDF-15	M. Barma et al. 2017 [93]
52.3 ± 7.1 (no hypertension) 55.2 ± 8.5 (hypertension)	↑ NO ↑ IL-6 ↑ TNF	Exercise intensity was measured by a heart rate monitor and maintained in a low-intensity range (50% and 70% of HR_max_) for 40 min (upper + lower limbs)	↓ NO ↓ IL-6 ↓ TNF	L. A. Da silva et al. 2018 [94]
22–70 years old (hypertension)	↑ SBP ↑ DBP ↔ TNF ↔ ICAM1 ↑ EDN1 ↑ NOS2	4 physical activity training sessions of 40 min a week (bike or jogging)	↓ SBP ↓ DBP ↔ TNF ↔ ICAM1 ↓ EDN1 ↓ NOS2	L. Ferrari et al. 2019 [95]

Notes: ↓ refers to significant decreased; ↑ refers to significant increased; ↔ refers to no change. CRP, C-reactive protein (CRP); HDL, high-density lipoprotein; LDL, low-density lipoprotein; IL-6, interleukin 6; sTNFR1, soluble tumor necrosis factor receptor 1; IL-1*β*, interleukin 1 beta; TNF*α*, tumor necrosis factor alpha; IL-8, interleukin 8; IL-25, interleukin 25; Adam17, A disintegrin and metalloprotease 17; SBP, systolic blood pressure; DBP, diastolic blood pressure; ICAM1, intercellular adhesion molecule 1; EDN1, endothelin 1; NOS2, nitric oxide synthase 2; NO, nitric oxide; GDF-15, growth/differentiation factor-15.

**Table 3:** The deleterious effects of exercise on cytokines in different age populations.

Age (years)	Exercise/physical activity	Deleterious of exercise/physical activity on cytokine	References
22 ± 3	Exercise with high intensity (80–90% VO_2max_)	↑ IL-6, ↑ IL-10	A. J. Wadley et al. 2016 [96]
28.1 ± 3	Exercised in 3 groups for 24 h and performed 12 exercise blocks (4x cycling, 4x running, and 4x kayaking) with 46–63%. Each block consisted of 110-minute exercise followed by 10-minute rest for food intake	↑ IL-6, ↑ IL-8 ↑ CRP ↔ TNF-*α* ↔ IL-1*β*	P. Marklund et al. 2013 [97]
38.8 ± 10.6	Cycling bout for 2.1 hours (1.75-h preload + 10-km time trial combined (82.2 ± 6.1% HR_max_)	↑ IL-6, ↑ IL-10 ↑ TNF-*α* ↑ IL-1*β* ↑ IL-8	D. C. Nieman, 2019, [98]
39.1 ± 2.2	3 races: a 10 km race (10 km: 89.12% VO_2max_), a half-marathon (HM: 81.50% VO_2max_), and a marathon (M: 68.70% VO_2max_)	Increased cytokine levels in half marathon and marathon: ↑ IL-6, ↑ IL-8, ↑ IL-10, ↑ CRP	D. Gonzalo-Calvo et al. 2015 [99]
18–40 years old	Exercise with 70% VO2_max_, 87.8% HR_max_ (The workload was increased each 5 minutes with 25 watts.)	↑ IL-6, ↑ IL-10 ↑ TNF-*α*	S. M. Ulven et al. 2015 [100]
≥60 years old (overweight)	Combined weight training and walking 1 hour, 3 times a week for 18 months	↔ sTNF-R1 ↔ CRP ↔ IL-6	B. J. Nicklas et al. 2004 [101]
65–80 years	Regular exercise training for 6 months, progressive resistance strength training for 12 weeks	↔ TNF-*α* ↔ CRP ↔ IL-6	C. J. K. Hammett et al. 2004 [102], L. C. Rall et al. 1996 [103]

Notes: ↓ refers to significant decreased; ↑ refers to significant increased; ↔ refers to no change. IL-6, interleukin 6; IL-10, interleukin 10; IL-8, interleukin 8; CRP, C-reactive protein (CRP); TNF-*α*, tumor necrosis factor alpha; IL-1*β*, interleukin 1 beta; sTNFR1, soluble tumor necrosis factor receptor 1.

## References

[1] Kyurkchiev D., Bochev I., Ivanova-Todorova E. (2014). Secretion of immunoregulatory cytokines by mesenchymal stem cells. *World Journal of Stem Cells*.

[2] Fuentes E., Fuentes M., Alarcon M., Palomo I. (2017). Immune system dysfunction in the elderly. *Anais da Academia Brasileira de Ciências*.

[3] Rea I. M., Gibson D. S., McGilligan V., McNerlan S. E., Alexander H. D., Ross O. A. (2018). Age and age-related diseases: role of inflammation triggers and cytokines. *Frontiers in Immunology*.

[4] Bogdanis G. C. (2012). Effects of physical activity and inactivity on muscle fatigue. *Frontiers in Physiology*.

[5] Pedersen B. K., Bruunsgaard H., Ostrowski K. (2000). Cytokines in aging and exercise. *International Journal of Sports Medicine*.

[6] Kany S., Vollrath J. T., Relja B. (2019). Cytokines in inflammatory disease. *International Journal of Molecular Sciences*.

[7] Hojman P., Gehl J., Christensen J. F., Pedersen B. K. (2018). Molecular mechanisms linking exercise to cancer prevention and treatment. *Cell Metabolism*.

[8] Warburton D. E. R., Nicol C. W., Bredin S. S. (2006). Health benefits of physical activity: the evidence. *Canadian Medical Association Journal*.

[9] Booth F. W., Roberts C. K., Laye M. J. (2012). Lack of exercise is a major cause of chronic diseases. *Comprehensive Physiology*.

[10] Senchina D. S., Kohut M. L. (2007). Immunological outcomes of exercise in older adults. *Clinical Interventions in Aging*.

[11] Malaguarnera L., Cristaldi E., Vinci M., Malaguarnera M. (2007). The role of exercise on the innate immunity of the elderly. *European Review of Aging and Physical Activity*.

[12] Madermott A. Y., Mernitz H. (2006). Exercise and older patients: prescribing guidelines. *American Family Physician*.

[13] Füzéki E., Banzer W. (2018). Physical activity recommendations for health and beyond in currently inactive populations. *International Journal of Environmental Research and Public Health*.

[14] Munnings F. (1993). Strength training: for the young. *The Physician and SportsMedicine*.

[15] Hunter G. R., McCarthy J. P., Bamman M. M. (2004). Effects of resistance training on older adults. *Sports Medicine*.

[16] Fragala M. S., Cadore E. L., Dorgo S. (2019). Resistance training for older adults. *The Journal of Strength & Conditioning Research*.

[17] Williams M. A., Haskell W. L., Ades P. A. (2007). Resistance exercise in individuals with and without cardiovascular disease: 2007 update: a scientific statement from the American heart association council on clinical cardiology and council on nutrition, physical activity, and metabolism. *Circulation*.

[18] Hasegawa H., Mizoguchi I., Chiba Y. (2006). Expanding diversity in molecular structures and functions of the IL-6/IL-12 heterodimeric cytokine family. *Frontiers in Immunology*.

[19] Huang Y., Henry C. J., Dantzer R., Johnson R. W., Godbout J. P. (2008). Exaggerated sickness behavior and brain proinflammatory cytokine expression in aged mice in response to intracerebroventricular lipopolysaccharide. *Neurobiology of Aging*.

[20] Schulte W., Bernhagen J., Bucala R. (2013). Cytokines in sepsis: potent immunoregulators and potential therapeutic targets--an updated view. *Mediators of Inflammation*.

[21] Pedersen B. K., Febbraio M. A. (2008). Muscle as an endocrine organ: focus on muscle-derived interleukin-6. *Physiological Reviews*.

[22] Suzuki K., Nakaji S., Yamada M., Totsuka M., Sato K., Sugawara K. (2000). Systemic inflammatory response to exhaustive exercise: cytokine kinetics. *Exercise Immunology Review*.

[23] Feldmann M., Maini R. N. (2003). Lasker clinical medical research award. TNF defined as a therapeutic target for rheumatoid arthritis and other autoimmune diseases. *Nature Medicine*.

[24] Feldman A. M., Combes A., Wagner D. (2000). The role of tumor necrosis factor in the pathophysiology of heart failure. *Journal of the American College of Cardiology*.

[25] Tarkowski E., Liljeroth A.-M., Minthon L., Tarkowski A., Wallin A., Blennow K. (2003). Cerebral pattern of pro- and anti-inflammatory cytokines in dementias. *Brain Research Bulletin*.

[26] Laws S. M., Perneczky R., Wagenpfeil S. (2005). TNF polymorphisms in Alzheimer disease and functional implications on CSF beta-amyloid levels. *Human Mutation*.

[27] Ershler W. B. (2003). Biological interactions of aging and anemia: a focus on cytokines. *Journal of the American Geriatrics Society*.

[28] Ershler W. B., Keller E. T. (2000). Age-associated increased interleukin-6 gene expression, late-life diseases, and frailty. *Annual Review of Medicine*.

[29] Grosicki G. J., Barrett B. B., Englund D. A. (2020). Circulating interleukin-6 is associated with skeletal muscle strength, quality, and functional adaptation with exercise training in mobility-limited older adults. *The Journal of frailty & aging*.

[30] Krabbe K. S., Pedersen M., Bruunsgaard H. (2004). Inflammatory mediators in the elderly. *Experimental Gerontology*.

[31] Morley J. E., Baumgartner R. N. (2004). Cytokine-related aging process. *The Journals of Gerontology Series A: Biological Sciences and Medical Sciences*.

[32] Wei Z.-M., Laby R. J., Zumoff C. H. (1992). Harpin, elicitor of the hypersensitive response produced by the plant pathogen Erwinia amylovora. *Science*.

[33] Banks W. A., Morley J. E. (2003). Memories are made of this: recent advances in understanding cognitive impairments and dementia. *The Journals of Gerontology, Series A: Biological Sciences*.

[34] Siddappaji K. K., Gopal S., Gopal S. (2021). Molecular mechanisms in Alzheimer’s disease and the impact of physical exercise with advancements in therapeutic approaches. *AIMS Neuroscience*.

[35] Rubio-Perez J. M., Morillas-Ruiz J. M. (2012). A review: inflammatory process in Alzheimer’s disease, role of cytokines. *The Scientific World Journal*.

[36] Wang W. Y., Tan M. S., Yu J. T., Tan L. (2015). Role of pro-inflammatory cytokines released from microglia in Alzheimer’s disease. *Annals of Translational Medicine*.

[37] Steen E. H., Wang X., Balaji S., Butte M. J., Bollyky P. L., Keswani S. G. (2020). The role of the anti-inflammatory cytokine interleukin-10 in tissue fibrosis. *Advances in Wound Care*.

[38] Mhyre T. R., Boyd J. T., Hamill R. W., Maguire-Zeissuka K. A., Sato K., Sugawara K. (2021). Parkinson’s disease. *Subcellular Biochemistry*.

[39] Wang Q., Liu Y., Zhou J. (2015). Neuroinflammation in Parkinson’s disease and its potential as therapeutic target. *Translational Neurodegeneration*.

[40] Tansey M. G., Goldberg M. S. (2010). Neuroinflammation in Parkinson’s disease: its role in neuronal death and implications for therapeutic intervention. *Neurobiology of Disease*.

[41] Yan J., Fu Q., Cheng L. (2014). Inflammatory response in Parkinson’s disease (Review). *Molecular Medicine Reports*.

[42] Koprich J. B., Reske-Nielsen C., Mithal P., Isacson O. (2008). Neuroinflammation mediated by IL-1beta increases susceptibility of dopamine neurons to degeneration in an animal model of Parkinson’s disease. *Journal of Neuroinflammation*.

[43] Scalzo P., Kümmer A., Cardoso F., Teixeira A. L. (2010). Serum levels of interleukin-6 are elevated in patients with Parkinson’s disease and correlate with physical performance. *Neuroscience Letters*.

[44] Bolton C., Smith P. A. (2015). Defining and regulating acute inflammatory lesion formation during the pathogenesis of multiple sclerosis and experimental autoimmune encephalomyelitis. *CNS & Neurological Disorders - Drug Targets*.

[45] Bolton C. (2021). An evaluation of the recognised systemic inflammatory biomarkers of chronic sub-optimal inflammation provides evidence for inflammageing (IFA) during multiple sclerosis (MS). *Immunity & Ageing*.

[46] Goverman J. M. (2012). Immune tolerance in multiple sclerosis. *Immunological Reviews*.

[47] Riedhammer C., Weissert R. (2015). Antigen presentation, autoantigens, and immune regulation in multiple sclerosis and other autoimmune diseases. *Frontiers in Immunology*.

[48] Massey J. C., Sutton I. J., Ma D. D. F., Moore J. J. (2018). Regenerating immunotolerance in multiple sclerosis with autologous hematopoietic stem cell transplant. *Frontiers in Immunology*.

[49] Murray T. J. (2005). Multiple sclerosis: the history of a disease. *Journal of the Royal Society of Medicine*.

[50] Monastero R. N., Pentyala S. (2017). Cytokines as biomarkers and their respective clinical cutoff levels. *International Journal of Inflammation*.

[51] Chung H. Y., Kim D. H., Lee E. K. (2019). Redefining chronic inflammation in aging and age-related diseases: proposal of the senoinflammation concept. *Aging and Disease*.

[52] Godbout J. P., Chen J., Abraham J. (2005). Exaggerated neuroinflammation and sickness behavior in aged mice following activation of the peripheral innate immune system. *The FASEB Journal*.

[53] McPhee J. S., French D. P., Jackson D., Nazroo J., Pendleton N., Degens H. (2016). Physical activity in older age: perspectives for healthy ageing and frailty. *Biogerontology*.

[54] Prince M. J., Wu F., Guo Y. (2015). The burden of disease in older people and implications for health policy and practice. *The Lancet*.

[55] Huntula S., Saegusa H., Wang X., Zong S., Tanabe T. (2019). Involvement of N-type Ca^2+^ channel in microglial activation and its implications to aging-induced exaggerated cytokine response. *Cell Calcium*.

[56] Tan L. F., Lim Z. Y., Choe R., Seetharaman S., Merchant R. (2017). Screening for frailty and sarcopenia among older persons in medical outpatient clinics and its associations with healthcare burden. *Journal of the American Medical Directors Association*.

[57] Elward K., Larson E. B. (1992). Benefits of exercise for older adults: a review of existing evidence and current recommendations for the general population. *Clinics in Geriatric Medicine*.

[58] Li J., Siegrist J. (2012). Physical activity and risk of cardiovascular disease-a meta-analysis of prospective cohort studies. *International Journal of Environmental Research and Public Health*.

[59] Thompson P. D., Buchner D., Pina I. L. (2003). Exercise and physical activity in the prevention and treatment of atherosclerotic cardiovascular disease: a statement from the council on clinical cardiology (subcommittee on exercise, rehabilitation, and prevention) and the council on nutrition, physical activity, and metabolism (subcommittee on physical activity). *Circulation*.

[60] Farmer J., Zhao X., van Praag H., Wodtke K., Gage F. H., Christie B. R. (2004). Effects of voluntary exercise on synaptic plasticity and gene expression in the dentate gyrus of adult-male Sprague-Dawley rats in vivo. *Neuroscience*.

[61] Franzke B., Halper B., Hofmann M. (2015). The effect of six months of elastic band resistance training, nutritional supplementation or cognitive training on chromosomal damage in institutionalized elderly. *Experimental Gerontology*.

[62] Kohut M. L., McCann D. A., Russell D. W. (2006). Aerobic exercise, but not flexibility/resistance exercise, reduces serum IL-18, CRP, and IL-6 independent of beta-blockers, BMI, and psychosocial factors in older adults. *Brain, Behavior, and Immunity*.

[63] Nicklas B. J., Hsu F. C., Brinkley T. J. (2008). Exercise training and plasma C-reactive protein and interleukin-6 in elderly people. *Journal of the American Geriatrics Society*.

[64] Janssen I. (2006). Influence of sarcopenia on the development of physical disability: the Cardiovascular Health Study. *Journal of the American Geriatrics Society*.

[65] Kamel H. K. (2003). Sarcopenia and aging. *Nutrition Reviews*.

[66] Morley J. E., Abbatecola A. M., Argiles J. M. (2011). Sarcopenia with limited mobility: an international consensus. *Journal of the American Medical Directors Association*.

[67] North B. J., Sinclair D. A. (2012). The intersection between aging and cardiovascular disease. *Circulation Research*.

[68] Fiuza-Luces C., Santos-Lozano A., Joyner M. (2018). Exercise benefits in cardiovascular disease: beyond attenuation of traditional risk factors. *Nature Reviews Cardiology*.

[69] Miller T. D., Balady G. J., Fletcher G. F. (1997). Exercise and its role in the prevention and rehabilitation of cardiovascular disease. *The Society of Behavioral Medicine*.

[70] Song X. A., Jia L. L., Cui W. (2014). Inhibition of TNF-alpha in hypothalamic paraventricular nucleus attenuates hypertension and cardiac hypertrophy by inhibiting neurohormonal excitation in spontaneously hypertensive rats. *Toxicology and Applied Pharmacology*.

[71] Shi Z., Gan X. B., Fan Z. D. (2011). Inflammatory cytokines in paraventricular nucleus modulate sympathetic activity and cardiac sympathetic afferent reflex in rats. *Acta Physiologica*.

[72] Paffenbarger R. S., Wing A. L., Hyde R. T., Jung D. L. (1983). Physical activity and incidence of hypertension in college alumni. *American Journal of Epidemiology*.

[73] Kaplan N. M. (1991). Long-term effectiveness of nonpharmacological treatment of hypertension. *Hypertension*.

[74] Nogueira-Ferreira R., Moreira-Goncalves D., Silva A. F. (2016). Exercise preconditioning prevents MCT-induced right ventricle remodeling through the regulation of TNF superfamily cytokines. *International Journal of Cardiology*.

[75] Jia L. L., Kang Y. M., Wang F. X. (2014). Exercise training attenuates hypertension and cardiac hypertrophy by modulating neurotransmitters and cytokines in hypothalamic paraventricular nucleus. *PLoS One*.

[76] Gordon N. F., Scott C. B., Wilkinson W. J., Duncan J. J., Blair S. N. (1990). Exercise and mild essential hypertension. Recommendations for adults. *Sports Medicine*.

[77] Church T. S., Kampert J. B., Gibbons L. W., Barlow C. E., Blair S. N. (2001). Usefulness of cardiorespiratory fitness as a predictor of all- cause and cardiovascular disease mortality in men with systemic hypertension. *The American Journal of Cardiology*.

[78] Evenson K. R., Stevens J., Thomas R., Cai J. (2004). Effect of cardiorespiratory fitness on mortality among hypertensive and normotensive women and men. *Epidemiology*.

[79] Brandon L. J., Sharon B. F., Boyette L. W. (1997). Effects of a four- month strength training program on blood pressure in older adults. *The Journal of Nutrition, Health & Aging*.

[80] Taaffe D. R., Harris T. B., Ferrucci L., Rowe J., Seeman T. E. (2000). Cross-sectional and prospective relationships of interleukin-6 and C-reactive protein with physical performance in elderly persons: MacArthur studies of successful aging. *The Journals Of Gerontology. Series A, Biological Sciences and Medical Sciences*.

[81] Reuben D. B., Judd-Hamilton L., Harris T. B., Seeman T. E., MacArthur Studies of Successful Aging (2003). The associations between physical activity and inflammatory markers in high-functioning older persons: macarthur studies of successful aging. *Journal of the American Geriatrics Society*.

[82] Pischon T., Hamkinson S. E., Hotamisligil G. S., Rifai N., Rimm E. B. (2003). Leisure-time physical activity and reduced plasma levels of obesity-related inflammatory markers. *Obesity Research*.

[83] Colbert L. H., Visser M., Simonsick E. M. (2004). Physical activity, exercise, and inflammatory markers in older adults: findings from the health, aging and body composition study. *Journal of the American Geriatrics Society*.

[84] Pitsavos C., Panagiotakos D. B., Chrysohoou C., Kavouras S., Stefanadis C. (2005). The associations between physical activity, inflammation, and coagulation markers, in people with metabolic syndrome: the ATTICA study. *European Journal of Cardiovascular Prevention & Rehabilitation*.

[85] Rahimi K., Secknus M. A., Adam M. (2005). Correlation of exercise capacity with high-sensitive C-reactive protein in patients with stable coronary artery disease. *American Heart Journal*.

[86] Arsenault B. J., Cartier A., Cote M. (2009). Body composition, cardiorespiratory fitness, and low-grade inflammation in middle-aged men and women. *The American Journal of Cardiology*.

[87] Beavers K. M., Brinkley T. E., Nicklas B. J. (2010). Effect of exercise training on chronic inflammation. *Clinica Chimica Acta*.

[88] Sardeli A. V., Tomeleri C. M., Cyrino E. S., Fernhall B., Cavaglieri C. R., Chacon-Mikahil M. P. T. (2018). Effect of resistance training on inflammatory markers of older adults: a meta-analysis. *Experimental Gerontology*.

[89] Goldhammer E., Tanchilevitch A., Maor I., Beniamini Y., Rosenschein U., Sagiv M. (2005). Exercise training modulates cytokines activity in coronary heart disease patients. *International Journal of Cardiology*.

[90] Ribeiro-Samora G. A., Rabelo L. A., Ferreira A. C. C. (2017). Inflammation and oxidative stress in heart failure: effects of exercise intensity and duration. *Brazilian Journal of Medical and Biological Research*.

[91] Racca V., Torri A., Grati P. (2020). Inflammatory cytokines during cardiac rehabilitation after heart surgery and their association to postoperative atrial fibrillation. *Scientific Reports*.

[92] Lima L. G., Bonardi J. M., Campos G. O. (2015). Effect of aerobic training and aerobic and resistance training on the inflammatory status of hypertensive older adults. *Aging Clinical and Experimental Research*.

[93] Barma M., Khan F., Price R. J. G. (2017). Association between GDF-15 levels and changes in vascular and physical function in older patients with hypertension. *Aging Clinical and Experimental Research*.

[94] Da Silva L. A., Menguer L., Motta J. (2018). Effect of aquatic exercise on mental health, functional autonomy, and oxidative dysfunction in hypertensive adults. *Clinical and Experimental Hypertension*.

[95] Ferrari L., Vicenzi M., Tarantini L. (2019). Effects of physical exercise on endothelial function and DNA methylation. *International Journal of Environmental Research and Public Health*.

[96] Wadley A. J., Chen Y. W., Lip G. Y., Fisher J. P., Aldred S. (2016). Low volume-high intensity interval exercise elicits antioxidant and anti-inflammatory effects in humans. *Journal of Sports Sciences*.

[97] Marklund P., Mattsson C. M., Wahlin-Larsson B. (2013). Extensive inflammatory cell infiltration in human skeletal muscle in response to an ultraendurance exercise bout in experienced athletes. *Journal of Applied Physiology (1985)*.

[98] Nieman D. C., Wentz L. M. (2019). The compelling link between physical activity and the body’s defense system. *The Journal of Sport and Health Science*.

[99] de Gonzalo-Calvo D., Davalos A., Montero A. (2015). Circulating inflammatory miRNA signature in response to different doses of aerobic exercise. *Journal of Applied Physiology (1985)*.

[100] Ulven S. M., Foss S. S., Skjolsvik A. M. (2015). An acute bout of exercise modulate the inflammatory response in peripheral blood mononuclear cells in healthy young men. *Archives of Physiology and Biochemistry*.

[101] Nicklas B. J., Ambrosius W., Messier S. P. (2004). Diet-induced weight loss, exercise, and chronic inflammation in older, obese adults: a randomized controlled clinical trial. *The American Journal of Clinical Nutrition*.

[102] Hammett C. J. K., Oxenham H. C., Baldietal J. C. (2004). Effect of six months’ exercise training on C-reactive protein levels in healthy elderly subjects. *Journal of the American College of Cardiology*.

[103] Rall L. C., Roubenoff R., Cannon J. G., Abad L. W., Dinarello C. A., Meydani S. N. (1996). Effects of progressive resistance training on immune response in aging and chronic inflammation. *Medicine & Science in Sports & Exercise*.

